# Implementation of real-time PCR assays for diagnosing intestinal protozoa infections

**DOI:** 10.1007/s00436-025-08483-3

**Published:** 2025-04-08

**Authors:** Christian N. Lotz, Pierre H. H. Schneeberger, Maura Concu, Said M. Ali, Emmanuel C. Mrimi, Jennifer Keiser

**Affiliations:** 1 Swiss Tropical and Public Health Institute, Allschwil, Switzerland; 2 University of Basel, Basel, Switzerland; 3 Public Health Laboratory-Ivo de Carneri, Chake, Pemba, Tanzania; 4 Ifakara Health Institute, Ifakara, Tanzania

**Keywords:** Protozoa, *Chilomastix mesnili*, qPCR, Microscopy, Emodepside

## Abstract

Intestinal protozoa infections present a major public health challenge, particularly in areas with poor sanitation and limited access to clean water. Effective diagnostic methods are critical, yet traditional microscopy, though widely used for its simplicity, lacks the sensitivity and specificity of modern techniques like real-time Polymerase Chain Reaction (qPCR), making the latter a more effective tool for monitoring and assessing the burden of intestinal protozoa diseases. In this study, we implemented two duplex qPCR assays to detect *Entamoeba dispar* + *Entamoeba histolytica* and *Cryptosporidium* spp. + *Chilomastix mesnili*, along with singleplex assays for *Giardia duodenalis* and *Blastocystis* spp., using a 10 µL reaction volume. This marks the first molecular detection of *Chilomastix mesnili* by qPCR, enhancing diagnostic precision. Using these, we analyzed stool samples from 70 patients on Pemba Island, Tanzania, before and 54 samples after treatment with 20, 25, or 30 mg of emodepside or placebo, aiming to assess protozoa prevalence for this region and emodepside’s potential antiprotozoal effects. Our qPCR reliably detected protozoa in 74.4% of samples, with *Entamoeba histolytica* and *Entamoeba dispar* in 31.4% of cases. Notably, one-third of these infections were caused by *Entamoeba histolytica*. No significant reduction in protozoa was observed after emodepside treatment compared to placebo. The study highlights the utility of qPCR in providing species-level differentiation and improving the speed and cost-effectiveness of testing. The high prevalence of protozoa in this region underscores the need for continued monitoring and control efforts, though emodepside was not effective against protozoa infections.

**Supplementary Information:**

The online version contains supplementary material available at 10.1007/s00436-025-08483-3.

## Background

Intestinal protozoa infections are a major contributor to gastrointestinal morbidity, malnutrition, and increased global mortality (Fletcher et al. 2012; Gedle et al. 2017). They pose a significant public health burden, accounting for approximately 58 million cases of diarrhea annually (Putignani and Menichella 2010; Thompson and Ash 2016). Among the most prevalent protozoa are *Entamoeba histolytica*, *E. Entamoeba dispar*, *Cryptosporidium* spp., *Giardia duodenalis*, *Blastocystis* spp*.*, and *Chilomastix mesnili*.

*Entamoeba histolytica* causes amoebiasis, leading to 40,000–100,000 deaths annually, ranking second to malaria in morbidity and mortality for parasitic diseases (Stanley 2003). *Entamoeba dispar* is morphologically similar to *E. histolytica*, and despite causing sporadic cases of gastritis, it is considered a non-pathogenic commensal (Graffeo et al. 2014; Oliveira et al. 2015). Although molecular diagnostics have facilitated their distinction, their precise biological functions and interactions within the host remain poorly understood (Fitri et al. 2022; McHardy et al. 2014). Cryptosporidiosis and giardiasis are the most common protozoa diseases affecting children under five and immunocompromised individuals (Arapi et al. 2016; Hunter and Nichols 2002). But despite its high prevalence of 300 million detected cases annually, giardiasis only occasionally causes diarrheal diseases compared to cryptosporidiosis (Lanata et al. 2013). Just as common are *Blastocystis* infections, which can reach prevalence between 50 and 60% in developing countries (Duda et al. 2015). Most *Blastocystis* infections are asymptomatic and have even been associated with a healthy gut microbiome. However, the organism and its pathogenesis remain poorly understood (Duda et al. 2015). The same applies to *C. mesnili*. Although some reports suggest potential pathogenicity, it is generally considered non-pathogenic (Barnham 1977; Speich et al. 2013; Suzuki et al. 2023). Furthermore, with a prevalence of about 13% in developing countries, it is an important indicator of fecal contamination in food or water sources (CDC 2019; Speich et al. 2013).

Most of the disease burden data are obtained by traditional bright-field microscopy, which, due to its cost-effectiveness, is still often the primary method for diagnosis. This technique is characterized by challenges in sample preservation and technical limitations, such as the inability to distinguish morphologically identical species (Fitri et al. 2022; Hamzah et al. 2006; McHardy et al. 2014). Additionally, bright-field microscopy requires high-level expertise and labor-intensity, while readout is subjective (van Lieshout and Roestenberg 2015). Recently, real-time Polymerase Chain Reaction (qPCR) has emerged as a molecular diagnostic method for several intestinal protozoa, providing unbiased data generation. Moreover, qPCR can distinguish morphologically identical species and provide higher specificity and sensitivity compared to bright-field microscopy (Bergquist et al. 2009; Easton et al. 2016; Schär et al. 2013). However, implementation of qPCR diagnostic faces challenges related to primer and probe design due to a lack of genomic data of protozoa, and its applicability in low-resource settings is limited by laboratory infrastructure, logistical constrains, and a lack of trained health professionals (Karadbhajane et al. 2021). Therefore, further development of qPCRs for additional protozoa, along with broadening the diagnostic capacity and improving its economic viability through multiplexing and reducing the amount of reaction agents, are important factors accelerating the diagnosis of protozoa infections.

The development of novel treatments against protozoa is as crucial as establishing a robust framework for understanding and detecting these infections. Currently, nitroimidazoles are the standard of care for most intestinal protozoa, with additional options including amebicides and paromomycin, primarily employed for amoebic infections (Miyamoto and Eckmann 2015; Rossignol 2010). However, resistance against nitroimidazoles is rising, treatment options for *Cryptosporidium* infections are limited, and the available nitazoxanide used for non-immunocompromised individuals varies in its efficacy (Bansal et al. 2006; Diptyanusa and Sari 2021; Krakovka et al. 2022; Miyamoto and Eckmann 2015; Rossignol 2010; Shirley et al. 2018). In a resource-limited field of protozoa drug development, drug repurposing is a useful strategy (Zubair et al. 2024). Emodepside, the veterinary anthelminthic drug, might be a potential drug candidate, since it activates potassium channels in helminths, which are also present in protozoa (Bah et al. 2021; Jimenez and Mesones 2022; Martin et al. 2012; Prole and Marrion 2012; Steinmann et al. 2015).

In this study, we aimed at implementing qPCR methods for the detection of six protozoa, including two duplexed reactions and the first molecular detection of *C. mesnili* in humans. Next, we applied the novel methods, to a subset of frozen stool samples from a clinical Phase 2a trial on emodepside on Pemba Island, Tanzania, providing an insight on the prevalence of these infections and the potential curative effect of emodepside on protozoa infections.

## Material and methods

### Primer and probe design

The primer and probe sequences for *Blastocystis* spp., *Cryptosporidium* spp., *E. histolytica*, *E. dispar*, and *G. duodenalis* were kindly provided from the accredited Diagnostic Center, at the Swiss Tropical and Public Health Institute (Table 1) (Novati et al. 1996; Stensvold et al. 2012; Verweij et al. 2003). For *C. mesnili*, we used the approach described in Schneeberger et al. to identify suitable primers and probes (Schneeberger et al. 2017). Briefly, eight partial sequences for the small ribosomal subunit were retrieved from the National Center for Biotechnology Information database using Nucleotide Basic Local Alignment Search Tool (BLASTN) and checked for highly conserved regions. These regions were compared with the NCBI database to assess degrees of similarity to close relatives, excluding nonspecific sequence similarities. Ultimately, primer and probes were selected meeting the following criteria: GC content of approximately 50%, length between 20 and 24 bases, and an estimated melting temperature (T_M_) of ~ 58 °C (Table 1). These partial sequences were taken for an individual BLASTN search to confirm their uniqueness; this step was also applied to primer and probe-sequences obtained from Diagnostic Center, Swiss TPH (Supplementary file 1: Table S1). All primers and probes were synthesized at Microsynth (Balgach, Switzerland). The selection of the dyes and quenchers was based on the detection capabilities of the CFX Maestro™ (Bio-Rad Laboratories Inc., Hercules, CA, USA).

**Table 1:** Summary of the properties established qPCR reactions

Organism	*Blastocystis* spp.	*C. mesnili*	*Cryptosporidium* spp*.*	*E. dispar*	*E. histolytica*	*G. duodenalis*
Target	Small subunit ribosomal RNA gene	18S ribosomal RNA	Small subunit ribosomal RNA gene	18S ribosomal RNA gene	Small subunit ribosomal RNA gene	Small subunit ribosomal RNA gene
Forward primer	GGT CCG GTG AAC ACT TTG GAT TT	TGC CTT GTC TTT TTG TTA CCA TAA AGA	ACA TGG ATA ACC GTG GTA ATT CT	AGG ATT GGA TGA AAT TCA GAT GTA CA	AGG ATT GGA TGA AAT TCA GAT GTA CA	GCT GCG TCA CGC TGC TC
Reverse primer	CCT ACG GAA ACC TTG TTA CGA CTT CA	GTC TGA ACT GTT ATT CCA TAC TGC AA	CAA TAC CCT ACC GTC TAA AGC TG	TAA GTT TCA GCC TTG TGA CCA TAC	TAA GTT TCA GCC TTG TGA CCA TAC	GAC GGC TCA GGA CAA CGG T
c(primer) [uM]	0.3	0.5	0.5	0.5	0.5	0.5
Probe sequence	TCG TGT AAA TCT TAC CAT TTA GAG GA	GCA GGT CGT GCC CTT GTG G	ACT CGA CTT TAT GGA AGG GTT GTA T	TGA AGA AAC ATT GTT TCT AAA TCC AAG T	AGA GAA GCA TTG TTT CTA GAT CTG A	CCC GCG GCG GTC CCT GCT AG
c(probe) [uM]	0.25	0.25	0.25	0.25	0.25	0.25
Probe	Hex	FAM	Cy5	FAM	Cy5	Hex
Quencher	BHQ1	BHQ1	BHQ2	BHQ1	BHQ2	BHQ1
Annealing temp [°C]	57.9	60.6	60.6	58.9	58.9	63.4
RFU	150	750	750	400	700	800

# Depicted is the target of each primer and probe, with their working concentrations (c(primer), c(probe)) and corresponding annealing temperature (temp). Furthermore, the fluorophore at 5'- and the quencher at 3'end of the probes are named (*Cy5*; Cyanine 5, *FAM*; Fluorescein amidite, *HEX*; Hexachloro-fluorescein, *BHQ*; Black Hole Quencher) and the Relative Fluorescence Unit (RFU) that needs to be reached in the CFX Maestro to count as positive sample is displayed

### Development of protocols for real-time PCR assays

Primer and probe sequences were tested in duplicates using at least three independent stool samples, each confirmed previously for the presence of the respective protozoa through microscopy (Samples provided by the Diagnostic Center at Swiss TPH). Plasmids containing a 120–250 base pair insert with the primer and probe sequences, along with an ampicillin or kanamycin resistance gene, were simultaneously designed and ordered for each protozoa species from BioCat in Heidelberg, Germany. Cycle conditions, as well as primer and probe concentrations, were refined to optimize the signal-to-noise ratio based on the plasmid and the positive samples. Sensitivity for each reaction was determined through a calibration series using ten-fold dilution series of corresponding plasmids. The resulting cycle threshold (Ct) values, indicating significant amplification, were plotted against the base-10 logarithm of the related dilution series (Basuni et al. 2011).

The limit of quantification was established as the lowest Ct value, where the calibration still reached a sufficient cut-off signal and displayed a sigmoidal shape. To ensure high specificity, the single and duplexed reactions were tested on stool samples from non-infected female NMRI mice (Charles River, Germany) and microscopically negative samples from humans (Diagnostic Center, Swiss TPH). This testing was repeated after spiking these samples with the different plasmids.

Subsequently, detection of *E. dispar* + *E. histolytica* and *C. mesnili* + *Cryptosporidium* spp. was attempted in a single reaction. Both duplexed reactions were thoroughly tested with and without other targets to rule out DNA cross-reaction or inhibition between different primers, probes, or targets (see Supplementary file 2: Fig. S1). The human 16S mitochondrial rRNA is based on published information and served as internal amplification and DNA extraction control (Supplementary file 3: Table S2) (Le et al. 2022; Zendejas-Heredia et al. 2021). The probe was modified to be compatible with the TaqMan assay using the Texas red fluorophore at 5′- and the BHQ−2 at 3′end. The annealing temperature was adapted for the different protozoa and cross-reaction with other primers and probes were out ruled as described above.

For the qPCR reaction, 5 μL of the TaqMan Gene Expression Master Mix (ThermoFisher Switzerland) was mixed with the primers and probes (final concentration see Table 1). The solution was brought up to 8 μL using DNase-free water (Gibco Switzerland) and 2 μL of the samples or the controls were added, thoroughly mixed, and pipetted into an Armadillo High Performance 384-well plate (ThermoScientific, Switzerland). The final solution was amplified on the CFX Maestro™ following this program: initial pre-amplification for 2 min at 50 °C, followed by 10 min at 95 °C. The program was set for 50 amplification cycles consisting of 15 s at 95 °C and 1 min at the primer-specific annealing temperature (Table 1). To ensure accuracy, each sample was run in duplicate. Controls included two samples containing ultrapure water and standards containing 1.000 and 1.000.000 gene copy numbers/µL, for each species.

### Stool samples and ethics

Stool samples in this study were collected in the framework of a phase 2a randomized controlled trial aimed at evaluating the efficacy of emodepside against soil-transmitted helminth infections. Approval for the study was granted by the Zanzibar Ministry of Health (Ref: NO.ZAHREC/03/JUNE/2021/11), the Zanzibar Food and Drug Agency (1.0 V1.0; 08.10.2020), and the Ethics Committee Northwest and Central Switzerland (AO_2021-00028). The trials adhered to the principles outlined in the Declaration of Helsinki and followed the guidelines of Good Clinical Practice. Participants provided informed consent. The study is registered with ClinicalTrial.gov (NCT05017194) (Mrimi et al. 2023). For this study, samples were selected from participants receiving one of the three highest emodepside doses (20, 25, and 30 mg) or from the placebo treatment arm. Stool samples analyzed were collected 1 day prior to the treatment (*n* = 70) and 14 up to 21 days post-treatment (*n* = 54). Approximately, 500 µL of the stool sample was collected from each participant, stored at − 20 °C, and subsequently shipped to the Swiss TPH in Allschwil, Switzerland.

### DNA extraction

To extract protozoa DNA, approximately 150 mg per sample of frozen stool was processed using DNeasy PowerSoil Pro Kits (Qiagen; Hilden, Germany) and eluted in 60 µL elution buffer (C6). The DNA concentration was measured using a NanoDrop™ One/OneC (ThermoFisher, Switzerland), ensuring successful extraction (DNA concentration > 25 ng/µL and a 260/230 nm absorbance ratio of 2 ± 0.2). If these criteria were not met, the extraction was repeated.

### Data preparation for qPCR

Data cleaning and quality checking for the qPCR samples were done using the software CFX Maestro™. Stool samples were considered positive based on specific criteria. First, the amplification curve shape was required to be sigmoidal, indicating a reliable amplification pattern. Second, the fluorescence signal had to exceed a pre-established threshold, distinguishing it from background noise while preserving valid results. Additionally, this signal had to reach the threshold within a Ct range, as defined for each protozoa in Table 2. Following these criteria, the Ct values of the samples were transformed into gene copy numbers (GCN) per µL. This process ensured the accurate determination and validation of positive samples through qPCR.

**Table 2:** Summary of the calibration parameters

Organism	*Blastocystis* spp*.*	*C. mesnili*	*Cryptosporidium* spp.	*E. dispar*	*E. histolytica*	*G. duodenalis*
Calibration (log_10_(GCN))	(Ct-43.8)/ − 3.9	(Ct-44.6)/ − 3.7	(Ct-44.5)/ − 3.6	(Ct-42.8)/ − 3.4	(Ct-43.5)/ − 3.4	(Ct-43.1)/ − 3.5
*R*^2^-value	0.9962	0.9996	0.9993	0.9929	0.9938	0.992
Efficiency [%]	80.1	85.2	90.4	97.9	97.0	92.9
Dynamic range 2*10^X	0–7	1–9	0–9	1–8	0–8	0–8
LLOQ (GCN/µL)	2	20	2	20	2	2

Depicted are the individual calibration equations for GCN and their corresponding R^2^-values. From the calibration curve, there was also determined the doubling efficacy [%], the dynamic range and lowest limit of quantification (LLOQ) were samples can be quantified

### Statistical analysis

Statistical analysis was performed using R (Version 4.3.3). For the calibration curves, the obtained Ct values were applied against the common logarithm of the dilution and the least squares method was used to determine the linear equation. For prevalence calculation, the number of positive samples was divided by the total number of samples analyzed. The efficacy of emodepside against protozoa infection was assessed by comparing the infection status before and 3 weeks post-treatment (54 patients), utilizing Fisher’s exact test and a significance level of *p* < 0.05. The mean and its standard deviations were calculated using the “summarize()” function from the “dplyr” package in R.

## Results and discussion

### Testing and optimizing protozoa species-specific primers and probes

Each primer and probe (shown in Table 1) set was tested on at least two microscopy positive human stool samples for *E. dispar* and *E. histolytica*, as well as on three microscopy negative samples. The designed plasmids were used in parallel to optimize individual reactions regarding primer and probe concentrations, annealing temperature, and their Ct values, as shown in Table 1.

**Fig. 1:**
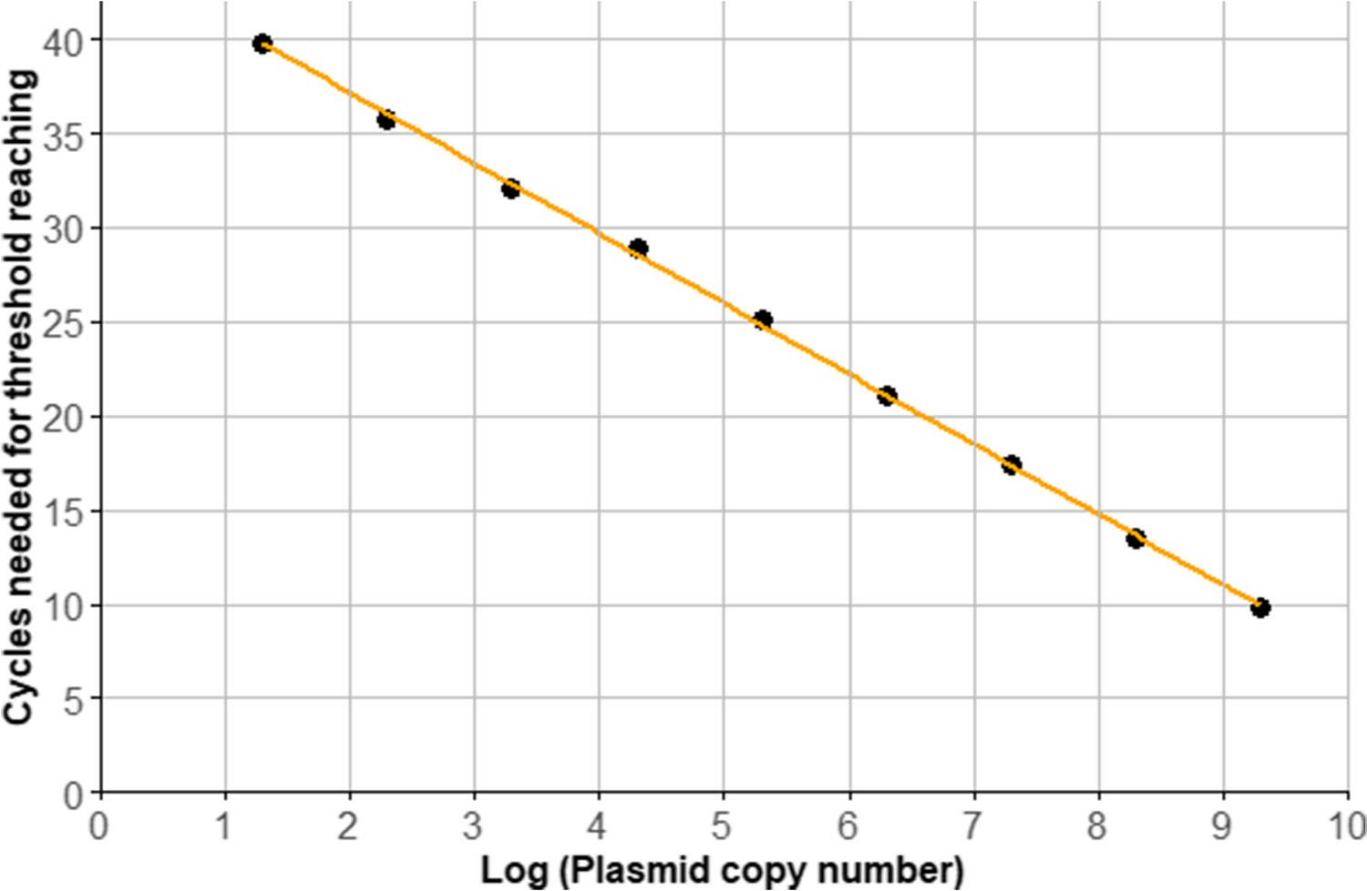
Calibration curve for the detection of *C. mesnili*. Created in R-Studio

After refining these parameters, no false negatives samples were detected, and all true positives were successfully identified. For generating quantitative results and a LLOQ, thus avoiding false positives, standard curves were established. First, the calibration curves were generated using water as the matrix. Next, DNA extracted from mouse stool was used. Finally, DNA from human stool was used for the calibration, as shown for *C. mesnili* in Fig. 1.

The calibration followed the Minimum Information for Publication of Quantitative Real-Time PCR Experiments guidelines. Table 2 shows the resulting linear equations, the *R*^2^-values for the calibration, their dynamic ranges, the LLOQ in GCN/µL, and the amplification efficiency, which needed to be between 80 and 110% (Azzopardi et al. 2021; Bustin et al. 2009; Nybo 2011).

Depicted are the individual calibration equations for GCN and their corresponding *R*^2^-values. From the calibration curve, there was also determined the doubling efficacy [%], the dynamic range and LLOQ where samples can be quantified.

Having samples tested positive via microscopy, as for example shown for *C. mesnili* in Supplementary file 2: Fig. S1, is important for estimating the qPCR signal intensity and sensitivity providing a reference point for the samples collected in the clinical trial. For *C. mesnili* (Supplementary file 2: Fig. S1), this is reached after approximately 21.54 cycles, translating into 162.57, GCN/µL, indicating that sensitivity and therefore the LLOQ will be a minor concern. Moreover, the Ct of its LLOQ is currently at ~ 39.82 (20 copies/µL), and therefore 32,51 times lower than the positive sample. This observation holds true across all reactions, where positive samples consistently exhibited concentrations at least 100 times higher than the LLOQ for the corresponding protozoa.

### Duplexing qPCR reactions

For economical and practical reasons, reactions were duplexed if applicable, limited by the different T_M_. The duplex calibrations of *Cryptosporidium* spp. + *C. mesnili* and *E. histolytica* + *E. dispar* were done as described above with human DNA as matrix spiked using both corresponding plasmids. Furthermore, cross-reaction and cross-inhibition were checked for primers, probes, and plasmids, depicted for *C. mesnili* in the Supplementary file 2: Fig. S1. For *E. dispar* and *E. histolytica*, as well as *C. mesnili* and *Cryptosporidium* spp., no effects could be seen. Due to the successful duplexing of protozoa pairs twice, the individual screening process for the six protozoa got 33% more efficient compared to singleplex reactions. Furthermore, reducing the overall sample volume to 10 µL compared to other studies makes these methods more suitable and practical for use in resource-limited settings (Keller et al. 2020; Menu et al. 2019).

### Prevalence and intensity of intestinal protozoa infection

**Fig. 2:**
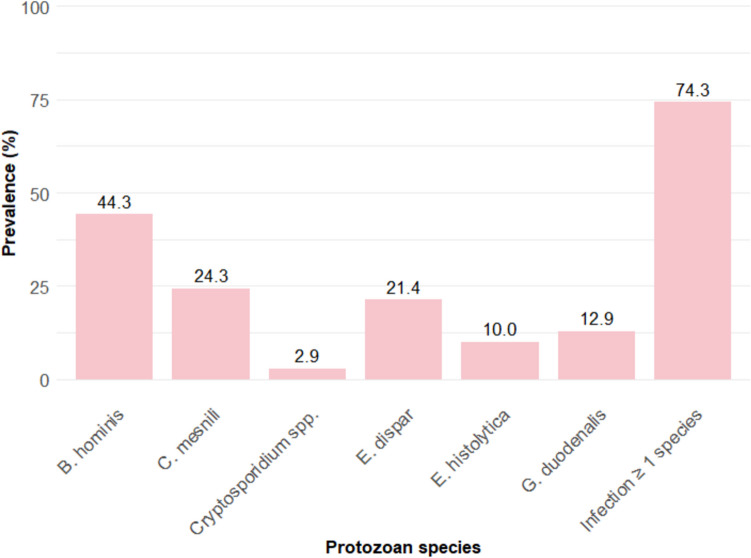
Observed percentage of infections of six different protozoa on Pemba Island as well as the percentage of people infected with at least one protozoa. Created in R-Studio

To understand the prevalence of protozoa infections on Pemba Island, we applied the established qPCR methods on a subset of 70 samples from patients enrolled in the emodepside clinical trial (Fig. 2).

Our findings indicate that 74.3% of the participants harbored at least one protozoa infection. Since this is the first study to investigate such a wide range of protozoa using qPCR within such a setting, no direct comparisons could be made (Hajissa et al. 2022). The prevalence of 44.3% *Blastocystis* infections aligns with findings conducted in related settings (Aguiar et al. 2007; Saksirisampant et al. 2003; Tan 2008). Given the requirement for study participants to be over 17, we observed a low incidence (2.9%) of *Cryptosporidium* spp. infection, which might be due to the developed immune systems and lower likelihood of exposure to contaminated water and environments compared to children (Checkley et al. 2015; Shrivastava et al. 2017). The prevalence of *E. histolytica/E. dispar* was found to be 31.4%. Through qPCR analysis, it was possible to discern that *E. histolytica*, the more pathogenic species, accounted for 31.9% of these infections. *Giardia duodenalis* prevalence was found to be lower when compared to studies conducted in similar settings (Barry et al. 2013; Belkessa et al. 2021; Speich et al. 2013). 24.3% of participants harbored an infection with *C. mesnili*, whereas the infection intensity varied (Supplementary file 4: Fig. S2). A prior study conducted on the same island in 2013 using microscopy reported a prevalence of *C. mesnili* of 12.9% (Speich et al. 2013). This difference of prevalence for *C. mesnili* is likely due to a higher sensitivity of qPCR compared to microscopy which is particularly pronounced in low prevalence settings such as industrialized countries (14.8% prevalence via qPCR vs. 1.1% via microscopy in a study conducted in Marseille) (Menu et al. 2019). These findings highlight the high prevalence of intestinal protozoa infections and the need for accurate detection, monitoring, and treatment, particularly in low-resource settings. They also emphasize the importance of qPCR diagnostics for disease elimination and the integration of protozoa surveillance into public health programs. Clinically, these results underscore the need for targeted therapies addressing the unique biology of protozoa.

### Efficacy of emodepside on intestinal protozoa infection

Furthermore, the study investigated whether emodepside affects the protozoa analyzed. This is based on the fact that emodepside activates a potassium channel in helminths, and the consideration of the vulnerability of protozoa to high internal potassium-cation levels (Bah et al. 2021; Jimenez and Mesones 2022; Martin et al. 2012; Prole and Marrion 2012; Steinmann et al. 2015). To explore this, samples from 54 patients were analyzed 1 day before and again 2–3 weeks after receiving either a placebo or emodepside (20 mg, 25 mg, or 30 mg) treatment. The post-treatment timeframe of 14–21 days was set according to standard guidelines for efficacy studies against helminths (Welsche et al. 2024). The sampling 1 day before treatment allows to minimize potential natural clearance (CDC 2021, van den Bijllaardt et al. 2014). The data (Supplementary file 4: Fig. S2 and Supplementary file 5: Table S3) show that treating patients with emodepside did not significantly clear protozoa. Additionally, we evaluated whether the intensity of protozoa infection was reduced, but no significant effect was observed, indicating that emodepside has no direct or indirect effect on intestinal protozoa (Supplementary file 5: Table S3). Supporting this, exploratory BLASTN searches did not identify close homologs for the SLO1 channel or the latrophilin channel in protozoan genomes, which are key targets of emodepside in helminths.

### Study limitations

One limitation of the study is that the samples used were not previously analyzed microscopically for protozoa, thus preventing direct comparisons. Using human 16S mitochondrial rRNA as an internal standard could introduce variability due to the high heterogeneity of stool samples. This and the preservation conditions may have affected the detection process (Esteva-Socias et al. 2020; Precioso et al. 2022). The 15–22-day follow-up period, tailored to the helminth efficacy trial, might be too long, as there is insufficient data on the residency time of protozoa in the gut and reinfection and hence distort the activity of emodepside on protozoa.

Also, a larger sample size would be beneficial to detect smaller trends and provide more statistical support for the findings. Moreover, the study population’s demographic characteristics can influence susceptibility to protozoa infections (Fletcher et al. 2014). Therefore, in vitro experiments with potential drug candidates are needed to lay the groundwork for tailored clinical trials that assess the efficacy of drugs against specific protozoa infections.

## Conclusion

In this work, we set up new and improved molecular diagnostic tools for protozoa infections. By developing the first molecular method for *C. mesnili* detection in humans, we expand our ability to monitor protozoa infections more comprehensively. Through multiplexing and efficient use of only 10 μL per qPCR reaction, a cost-effective method for detecting the most common and pathogenic intestinal protozoa was established. This tool holds promise to enhance future surveillance activities and facilitate the development of novel therapeutic interventions. Application of these diagnostic tools in a clinical trial cohort revealed that a tremendous number, 74.4%, of participants on Pemba Island harbored at least one protozoa infection. Knowing the global protozoa prevalences is fundamental when planning programs for reducing or eliminating potential pathogenic protozoa infections. Furthermore, we investigated whether the new promising anthelminthic emodepside affects protozoa infections. However, our findings revealed no discernible effects reinforcing emodepside’s targeted effectiveness against nematodes.

## Supplementary Information

Below is the link to the electronic supplementary material.

ESM 1 (DOCX 30.6 KB)

ESM 2 (PDF 93.2 KB)

ESM 3 (PNG 425 KB)

High Resolution Image (TIF 690 KB)

## Data Availability

No datasets were generated or analysed during the current study.
