# Supplementary material for: Implementation of real-time PCR assays for diagnosing intestinal protozoa infections
Source: Parasitol Res. 2025 Apr 8;124(4):40. doi: 10.1007/s00436-025-08483-3 (PMC11978536; doi:10.1007/s00436-025-08483-3)
Supplement: Supplementary file 1 — (DOCX 30.6 KB) [file 436_2025_8483_MOESM1_ESM.docx]

Supplementary information

# Supplementary tables and figure captions

## Supplementary file 1: Table S1: Outcomes from the Nucleotide Basic Local Alignment Search Tool (BLASTN) search encompassing each primer and probe of the protozoa species.

|  | Score | Expect | Identities | Gaps | % of correct hits |
| --- | --- | --- | --- | --- | --- |
| *Cryptosporidium* spp. |  |  |  |  |  |
| Forward Primer | 46.1 bits (23) | 0.044 | 23/23 | 0/23 | N/A |
| Reverse Primer | 46.1 bits (23) | 0.044 | 23/23 | 0/23 | 100 |
| Probe | 50.1 bits (25) | 0.004 | 25/25 | 0/25 | 100 |
| *C. mesnili* |  |  |  |  |  |
| Forward Primer | 54.0 bits (27) | 0.00004 | 27/27 | 0/27 | 21.8 |
| Reverse Primer | 52.0 bits (26) | 0.001 | 26/26 | 0/26 | 68.5 |
| Probe | 38.2 bits (19) | 11 | 19/19 | 0/19 | 52.5 |
| *E. dispar* |  |  |  |  |  |
| Forward Primer | 52.0 bits (26) | 0.001 | 26/26 | 0/26 | 79 |
| Reverse Primer | 48.1 bits (24) | 0.017 | 24/24 | 0/24 | 1.0 |
| Probe | 56.0 bits (28) | 0.0001 | 28/28 | 0/28 | 40.4 |
| *E. histolytica* |  |  |  |  |  |
| Forward Primer | 52.0 bits (26) | 0.001 | 26/26 | 0/26 | 18 |
| Reverse Primer | 48.1 bits (24) | 0.017 | 24/24 | 0/24 | 14.0 |
| Probe | 50.1 bits (25) | 0.004 | 25/25 | 0/25 | 64.1 |
| *G. duodenalis* |  |  |  |  |  |
| Forward Primer | 34.2 bits (17) | 169 | 17/17 | 0/17 | 78.8 |
| Reverse Primer | 38.2 bits (19) | 11 | 19/19 | 0/19 | 91.2 |
| Probe | 40.1 bits (20) | 2.7 | 20/20 | 0/20 | 100 |
| *Blastocystis* spp. |  |  |  |  |  |
| Forward Primer | 46.1 bits (23) | 0.044 | 23/23 | 0/23 | 100 |
| Reverse Primer | No significant accordance could be found | | | | |
| Probe | 52.0 bits (26) | 0.001 | 26/26 | 0/26 | 88.4 |

## Supplementary file 2, Fig. S1 Amplification curves of *C. mesnili* using qPCR. Created using the CFX Maestro^TM^ software

The Relative Fluorescence Unit (RFU) represents the measured fluorescence signal, displayed here for the FAM channel. All samples contain the C. mesnili primers and probe. All even-numbered samples contain on top the set for Cryptosporidium spp. Samples 1 and 2 contain 2 x 10^8^ plasmid copies/µL with the 18S ribosomal RNA insert; samples 5 and 6 contain 2 x 10 plasmid copies/µL. Samples 3 and 4 are microscopy-positive for C. mesnili. Samples 7 and 8 contain human DNA negative for C. mesnili, and samples 9 and 10 are negative controls containing 2 x 10^8^ plasmid copies/µL of Cryptosporidium spp..

## Supplementary file 3: Table S2: Properties of the internal standard used.

| Organism | Human |
| --- | --- |
| Target | Human mitochondrial genome |
| Forward primer | CGA CCT CGA TGT TGG ATC AG |
| Reverse primer | GAA CTC AGA TCA CGT AGG ACT TT |
| c(primer) [uM] | 0.166 |
| Probe sequence | CCC GAT GGT GCA GCC GCT ATT AAA |
| c(probe) [uM] | 0.166 |
| Probe | TXXRe |
| Quencher | BHQ2 |
| Annealing temp [°C] | 57.9-63.4 |
| Ct | 1000 |

# Working concentrations primer/ probe (c(primer), c(probe)), temp=temperature, Ct= cycle threshold

## Supplementary file 4, Fig. S2 Infection intensity of *C. mesnili* infections before and after treatment with emodepside (20, 25, 30 mg) or placebo. Created in R-Studio

## Supplementary file 5: Table S3: Effect on infection prevalence and infection intensity of emodepside on intestinal protozoa.

| Protozoan | Emodepside [mg] | | | Placebo |
| --- | --- | --- | --- | --- |
|  | 20 | 25 | 30 |  |
| Patients |  |  |  |  |
| Before treatment | 15 | 17 | 18 | 20 |
| After treatment | 11 | 13 | 13 | 17 |
| *Blastocystis* spp. |  |  |  |  |
| Mean GCN/ µl (SD) before treatment | 2503564 (5905894) | 134243938 (232416130) | 180369 (213193) | 8945789 (20545604) |
| Mean GCN/ µl (SD) after treatment | 24289442 (54211198) | 19173517 (33002843) | 1772633 (2737227) | 13042821 (25989631) |
| % of infected before treatment | 53.3 | 23.5 | 44.4 | 55.0 |
| % of infected after treatment | 45.5 | 23.1 | 46.2 | 47.1 |
| p value | 1.00 | 1.00 | 1.00 | 0.95 |
| Confidence intervals (95%) | 0.3-6.5 | 0.2-5.7 | 0.2-3.9 | 0.4-5 |
| Odds ratio | 1.4 | 1.0 | 0.9 | 1.4 |
| *Chilomastix mesnili* |  |  |  |  |
| Mean GCN/ µl (SD) before treatment | 2065875 (N/A) | 169063352 (337670082) | 136186455 (285292491) | 461395 (736441) |
| Mean GCN/ µl (SD) after treatment | 661673 (711652) | 1369665 (1960226) | 160354066 (282592298) | 32280305 (85959591) |
| % of infected before treatment | 13.3 | 29.4 | 33.3 | 20.0 |
| % of infected after treatment | 54.5 | 30.8 | 46.2 | 47.1 |
| p value | 0.09 | 1.00 | 0.75 | 0.26 |
| Confidence intervals | 0-0.9 | 0.2-4.5 | 0.1-2.5 | 0.1-1.2 |
| Odds ratio | 0.1 | 0.9 | 0.6 | 0.3 |
| *Cryptosporidium* spp. |  |  |  |  |
| Mean GCN/ µl (SD) before treatment | 0 (N/A) | 2.2 (N/A) | 0 (N/A) | 0 (N/A) |
| Mean GCN/ µl (SD) after treatment | 0 (N/A) | N/A (N/A) | 8.2 (N/A) | 0 (N/A) |
| % of infected before treatment | 0.0 | 11.8 | 0.0 | 0.0 |
| % of infected after treatment | 9.1 | 0.0 | 7.7 | 0.0 |
| p value | 0.65 | 0.71 | 0.68 | 1.00 |
| Confidence intervals | 0.0-N/A | N/A | 0.0-N/A | N/A |
| Odds ratio | 0 | N/A | 0 | N/A |
| *Entamoeba dispar* |  |  |  |  |
| Mean GCN/ µl (SD) before treatment | 25882 (0) | 1114 (N/A) | 5076 (6836) | 25386 (38932) |
| Mean GCN/ µl (SD) after treatment | 0 (N/A) | 2536551 (N/A) | 19561 (N/A) | 340032 (718938) |
| % of infected before treatment | 13.3 | 17.6 | 27.8 | 25.0 |
| % of infected after treatment | 0.0 | 7.7 | 7.7 | 29.4 |
| p value | 0.70 | 0.78 | 0.43 | 1.00 |
| Confidence intervals | N/A | 0.2-28.1 | 0.5-45.4 | 0.2-3.4 |
| Odds ratio | N/A | 2.6 | 4.6 | 0.8 |
| *Entamoeba histolytica* |  |  |  |  |
| Mean GCN/ µl (SD) before treatment | 2.5 (N/A) | 2.5 (N/A) | 36.8 (48.8) | 716 (1237) |
| Mean GCN/ µl (SD) after treatment | 0.9 (0) | 1.0 (0.4) | 2 (0.50) | 1.1 (N/A) |
| % of infected before treatment | 6.7 | 5.9 | 11.1 | 15.0 |
| % of infected after treatment | 9.1 | 23.1 | 15.4 | 5.9 |
| p value | 1.00 | 0.42 | 1.00 | 0.80 |
| Confidence intervals | 0-12.8 | 0-2.3 | 0.1-5.6 | 0.3-30 |
| Odds ratio | 0.7 | 0.2 | 0.7 | 2.8 |
| *Giardia intestinalis* |  |  |  |  |
| Mean GCN/ µl (SD) before treatment | 7402930.9 (10149126) | 193330 (270584) | 19733 (29178) | 71856 (101529) |
| Mean GCN/ µl (SD) after treatment | 25957758 (36709452) | 71852313 (N/A) | 929784 (1312990) | 428 (494) |
| % of infected before treatment | 14.3 | 33.3 | 0.0 | 0.0 |
| % of infected after treatment | 18.2 | 7.7 | 15.4 | 11.8 |
| p value | 1.00 | 1.00 | 1.00 | 1.00 |
| Confidence intervals | 0.1-5.9 | 0.1-19.8 | 0.2-7.7 | 0.1-6.6 |
| Odds ratio | 0.7 | 1.6 | 1.1 | 0.8 |
| Accumulated |  |  |  |  |
| Mean GCN/ µl (SD) before treatment | 2399651 (8027510) | 50583623 (190118932) | 27278334 (57108349) | 1901028 (4284749) |
| Mean GCN/ µl (SD) after treatment | 12727218 (30544101) | 18986409 (11654357) | 27179342 (71660629) | 9132717 (28167163) |
| % of infected before treatment | 80.0 | 58.8 | 77.8 | 80.0 |
| % of infected after treatment | 90.9 | 69.2 | 76.9 | 76.5 |
| p value | 0.84 | 0.89 | 1.00 | 1.00 |
| Confidence intervals | 0-4.5 | 0.1-2.9 | 0.2-5.8 | 0.3-5.9 |
| Odds ratio | 0.4 | 0.6 | 1.1 | 1.2 |

# GCN= Gene copy number, SD= Standard deviation
