# Supplementary figures and images for: Implementation of real-time PCR assays for diagnosing intestinal protozoa infections
Source: Parasitol Res. 2025 Apr 8;124(4):40. doi: 10.1007/s00436-025-08483-3 (PMC11978536; doi:10.1007/s00436-025-08483-3)

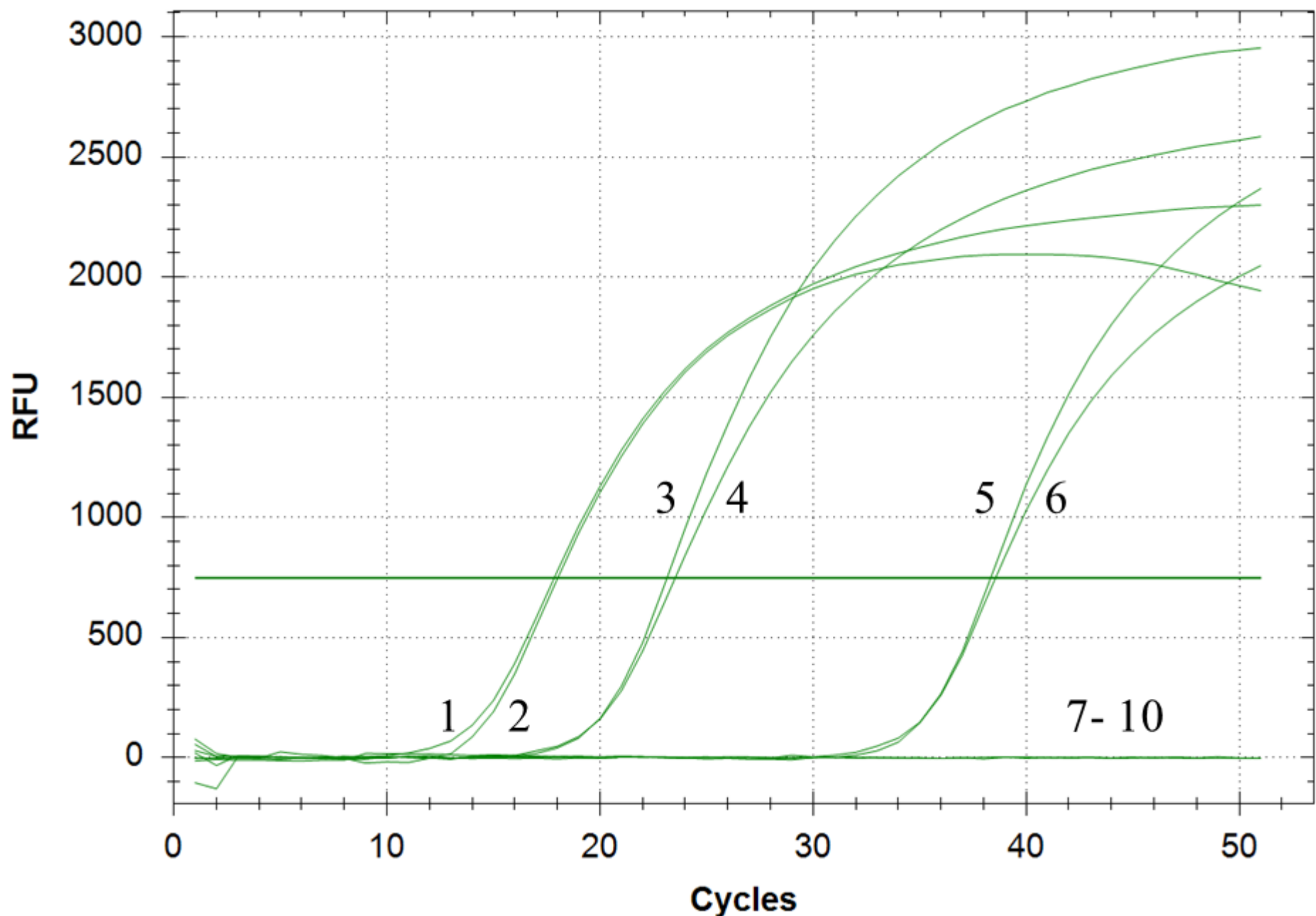

Supplement: Supplementary file 2 — (PDF 93.2 KB) [file 436_2025_8483_MOESM2_ESM.pdf]

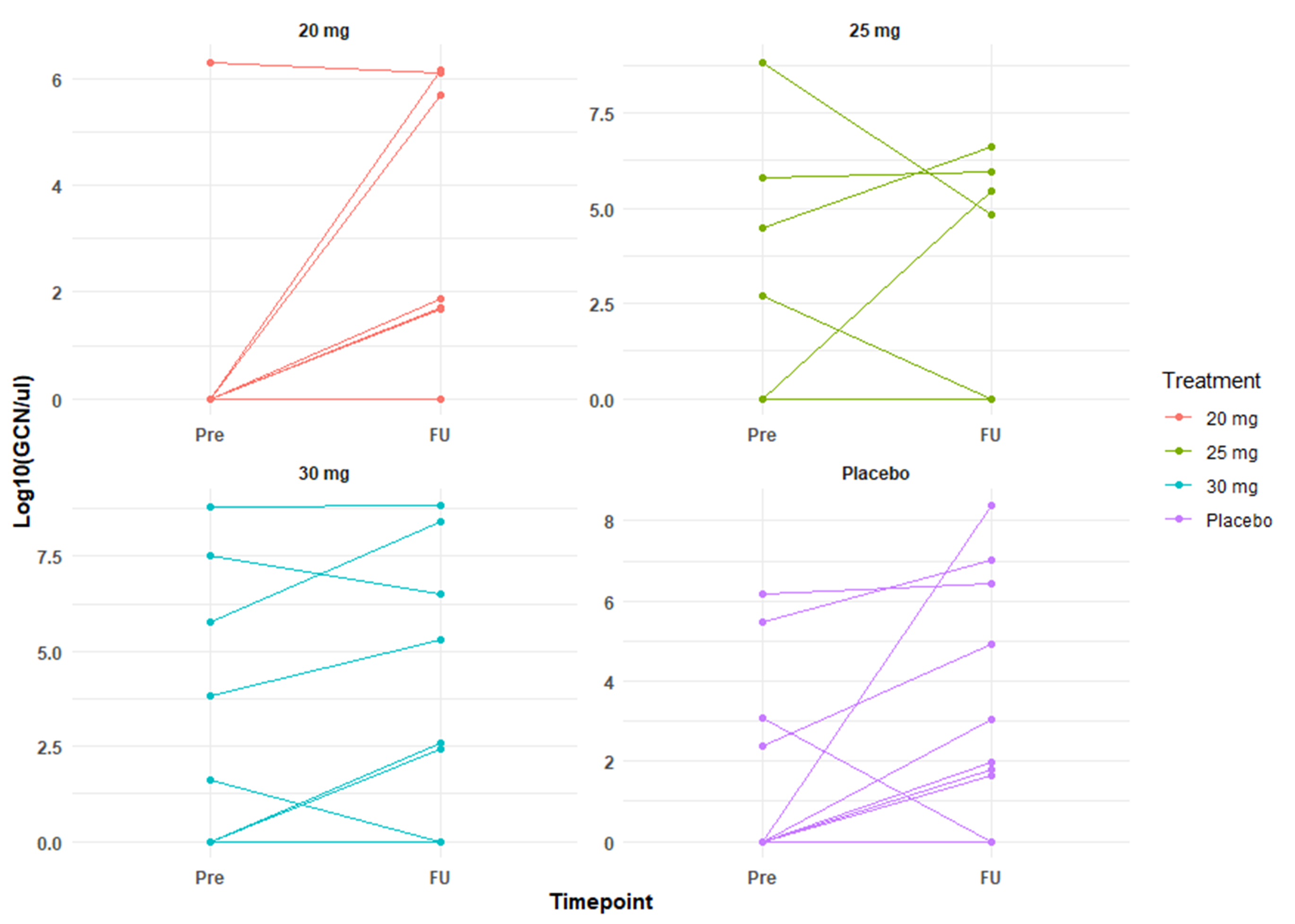

Supplement: Supplementary file 3 — (PNG 425 KB) [file 436_2025_8483_Fig3_ESM.png]

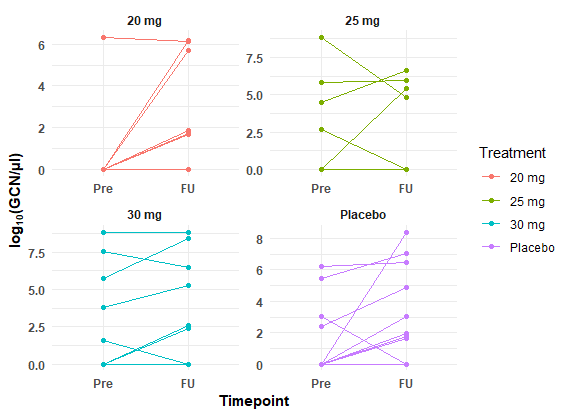

Supplement: Supplementary file 4 — High Resolution Image (TIF 690 KB) [file 436_2025_8483_MOESM3_ESM.tiff]
